# New Cellular Treatment Factor (NCTF) and Polydeoxyribonucleotide (VAMP) Injections Enhance Skin Regeneration: Experimental and Histological Evidence

**DOI:** 10.1111/jocd.70733

**Published:** 2026-02-11

**Authors:** Noury Adel, Jack Kolenda

**Affiliations:** ^1^ Oral and Maxillofacial Surgery Specialist Private Practice Cairo Egypt; ^2^ Department of Otolaryngology Head and Neck Surgery University of Toronto Toronto Ontario Canada

**Keywords:** angiogenesis, injectable therapy, NCTF, regenerative medicine, skin wound healing, VAMP

## Abstract

**Background:**

Optimizing cutaneous wound healing remains a priority in dermatology and plastic surgery. Bioactive injectables such as NCTF and VAMP are gaining interest for their potential to enhance angiogenesis and tissue regeneration, yet their combined effects remain underexplored.

**Objective:**

To compare the effects of NCTF, VAMP, and their combination on full thickness skin wound healing in an experimental hamster model, using histological and immunohistochemical analyses.

**Methods:**

One hundred forty‐four adult hamsters were randomly assigned to four groups: (1) control (full thickness 2 cm length & 2 mm width dorsal incision, no injection), (2) NCTF (0.1 mL), (3) VAMP (0.1 mL), and (4) combined NCTF + VAMP (0.05 mL each). Wounds were evaluated at baseline, day 3, day 7, and day 14. Tissue samples underwent hematoxylin–eosin (HE) and Masson's trichrome (MT) staining for histological assessment, and immunohistochemical staining for CD34 was performed to evaluate angiogenesis and capillary density.

**Results:**

All treatment groups showed faster wound closure, greater collagen deposition, and higher microvessel density than controls. The combination therapy produced the most pronounced improvements, followed by NCTF alone, then VAMP alone, with the control group showing the slowest healing. CD34 positive capillary density and collagen fiber alignment were significantly superior in the combination group at days 7 and 14 (*p* < 0.05).

**Conclusion:**

NCTF and VAMP both enhance full thickness skin wound healing, with the combination achieving the greatest histological and angiogenic improvements. These findings pertain to early and intermediate phases of wound healing (up to 14 days) and suggest potential benefit of combination therapy, pending further studies in larger animal models and clinical trials.

## 1 Introduction

The restoration of skin integrity after injury is a complex, highly coordinated process involving inflammation, cellular proliferation, angiogenesis, and extracellular matrix remodeling. While the skin possesses an inherent capacity for repair, full thickness wounds often heal slowly and may result in excessive scarring or compromised functional and aesthetic outcomes. In dermatology and plastic surgery, optimizing the quality and speed of wound healing remains a clinical priority, particularly for patients undergoing reconstructive procedures, trauma management, or aesthetic interventions [1, 2, 3, 4, 5].

Conventional wound care strategies, ranging from surgical closure techniques to topical dressings, can promote re epithelialization but do not actively modulate the cellular and vascular mechanisms essential for high quality tissue regeneration. As a result, there has been growing interest in bioactive injectable agents capable of enhancing fibroblast activity, stimulating angiogenesis, and regulating extracellular matrix deposition [6, 7, 8].

NCTF, or New Cellular Treatment Factor, is a polyrevitalizing injectable formulation developed for mesotherapy. It contains a balanced mixture of vitamins, amino acids, nucleotides, coenzymes, minerals, and hyaluronic acid. The hyaluronic acid component supports dermal hydration and provides a temporary scaffold for cellular migration, while the micronutrient complex facilitates fibroblast proliferation, collagen synthesis, and antioxidant protection. Experimental and clinical observations have suggested that NCTF enhances dermal regeneration by creating a metabolically favorable microenvironment for tissue repair [9, 10].

VAMP refers to a polydeoxyribonucleotide (PDRN) injectable preparation. PDRN is a biologically active mixture of DNA fragments, typically extracted from salmonid fish, with an average molecular weight ranging between 50 and 1500 kDa. It is known to act primarily through stimulation of A2A adenosine receptors, which enhances vascular endothelial growth factor (VEGF) expression and thereby promotes angiogenesis. In addition, PDRN supports fibroblast proliferation, stimulates collagen synthesis, and modulates inflammatory responses within the wound microenvironment. These properties make it a promising agent for accelerating tissue regeneration and improving the quality of skin repair in both experimental and clinical settings [11, 12, 13].

Previous experimental studies have demonstrated that PDRN alone can accelerate wound healing by enhancing angiogenesis and modulating inflammation in animal models. Similarly, polyrevitalizing solutions such as NCTF have been shown to improve dermal regeneration by promoting fibroblast proliferation and collagen synthesis when administered individually. However, these studies evaluated each agent in isolation, and no prior animal study has directly compared their effects or investigated whether a combined approach could provide synergistic benefits in full thickness skin wounds [9, 10, 11, 12, 13]. Our study addresses this gap by systematically examining both the individual and combined effects of NCTF and PDRN in a controlled hamster model.

The present study was designed to investigate, in a controlled experimental hamster model, the individual and combined effects of NCTF and VAMP on full thickness dorsal skin wound healing. It was hypothesized that the combination therapy would yield superior histological and angiogenic outcomes compared with either agent alone or untreated controls. Histological assessments using hematoxylin–eosin and Masson's trichrome staining and CD34 immunohistochemistry were employed to characterize epithelial regeneration, collagen deposition, and neovascularization at multiple time points during healing.

## 2 Materials and Methods

In this study, all procedures involving animals were performed with the highest regard for their welfare, following the ARRIVE guidelines and international ethical standards. The study protocol was approved by an Institutional Animal Care and Use Committee (IACUC), and every step was taken to minimize discomfort while using only the number of animals necessary to obtain scientifically valid results.

### 2.1 Animal Model and Justification

Adult male Syrian hamsters ( *Mesocricetus auratus* ) were chosen because their dorsal skin provides a sufficiently large and uniform surface for reproducible full thickness wounds, and their wound healing responses are well characterized in preclinical dermatologic and surgical research. While rodent healing involves more wound contraction than in humans, this model allows reliable histological and angiogenic evaluation before translating to larger animal or human studies.

### 2.2 Animal Characteristics, Acclimatization, Housing, and Diet

One hundred forty‐four adult male Syrian hamsters ( *Mesocricetus auratus* ), aged 8–10 weeks and weighing 100–140 g, were obtained from an accredited breeder. Animals were acclimatized for seven days before any procedures. Hamsters were housed in groups of two to three per polycarbonate cage with autoclaved bedding and environmental enrichment (nesting material and gnawing blocks). Room conditions were maintained at 20°C–24°C, relative humidity 45%–55%, with a 12:12‐h light: dark cycle. Standard pelleted rodent chow and filtered tap water were available ad libitum. Body weight and general health were recorded at baseline and at each sampling time point.

### 2.3 Sample Size and Allocation

This randomized, controlled, blinded study compared four treatment groups with terminal sampling at four time points (baseline, day 3, day 7, day 14). A priori power analysis, based on expected large effect sizes for histological and angiogenic end points from prior literature and pilot observations, indicated that *n* = 9 animals per group per time point would provide approximately 80% power to detect biologically meaningful differences at *α* = 0.05. Accordingly, 144 adult male Syrian hamsters were used and equally allocated to four groups (36 animals per group). Each group was sampled at four time points with *n* = 9 animals per group per time point.

The time points chosen in this study allowed evaluation of early inflammatory and proliferative phases of wound healing. The 14 day endpoint was selected to balance comprehensive histological assessment with animal welfare considerations, but it does not capture longer term remodeling or scar maturation.

### 2.4 Randomization and Blinding

Animals were randomly assigned to groups using a computer generated randomization schedule. Animals were identified by cage cards. Investigators responsible for macroscopic wound assessment, histological scoring, image analysis, and statistical analysis were blinded to group allocation until after data collection was complete.

### 2.5 Experimental Groups

- Group 1 (Control): full thickness linear dorsal skin incision (2 cm length, approximately 2 mm width), no injectable treatment (*n* = 36).
- Group 2 (NCTF): identical incision + single local injection of 0.1 mL NCTF (*n* = 36).
- Group 3 (VAMP/PDRN): identical incision + single local injection of 0.1 mL VAMP (PDRN containing product) (*n* = 36).
- Group 4 (Combination): identical incision + single local injection composed of 0.05 mL NCTF + 0.05 mL VAMP combined in one syringe (*n* = 36). Each group consisted of 36 animals (*n* = 9 per time point).

### 2.6 Anesthesia and Perioperative Care

General anesthesia was induced using an intraperitoneal injection of a ketamine–xylazine mixture, consisting of 0.03 mL ketamine (100 mg/mL; ~21.4 mg/kg) and 0.02 mL xylazine (20 mg/mL; ~2.9 mg/kg) per animal. Anesthetic depth was confirmed by the absence of pedal withdrawal and palpebral reflexes. The dorsal fur was shaved, and the skin was disinfected with povidone–iodine followed by 70% ethanol.

### 2.7 Wound Creation and Injectable Administration

Under sterile conditions, dorsal fur was clipped and the skin prepared with povidone–iodine followed by 70% ethanol. Using fine surgical scissors, a full thickness linear incision measuring 2 cm in length and approximately 2 mm in width was made along the mid dorsal line, penetrating the epidermis, dermis, and panniculus carnosus. Hemostasis was achieved by gentle pressure with sterile gauze. For treatment groups, the assigned injectable(s) were administered immediately after incision creation, distributed evenly along both wound edges using a BD insulin syringe. Control animals underwent identical handling but without injection. Incisions were left unsutured to heal by secondary intention.

### 2.8 Postoperative Care

Wounds were left uncovered to heal by secondary intention. For the first 48 h, animals were housed individually to prevent wound interference, then returned to their original cage grouping. Wound sites were inspected daily for infection, dehiscence, or other complications.

### 2.9 Evaluation Methods

Macroscopic wound photographs were taken at baseline, day 3, day 7, and day 14 using a standardized distance and lighting setup, with a metric scale included. Wound area was calculated using digital planimetry software, and percentage closure was determined relative to baseline. Epithelialization was graded on a 0–3 scale by two blinded observers.

At each designated time point, animals were euthanized by intraperitoneal injection of a lethal dose of ketamine hydrochloride (≥ 300 mg/kg), ensuring rapid and humane death. The wound site was excised en bloc using fine surgical scissors, together with a 1 mm margin of surrounding intact skin to include the wound edges in all specimens. The excision depth was standardized to capture the full thickness of the skin (epidermis, dermis, and panniculus carnosus).

To maintain uniformity, all tissue harvesting was performed by the same investigator using identical instruments and technique, and wound orientation was kept consistent across specimens. The number of animals sacrificed in each group at each time interval was identical (*n* = 9 per group per time point), ensuring balanced sampling and eliminating temporal or group‐related bias.

Excised tissue samples were immediately fixed in 10% neutral buffered formalin for at least 48 h, processed, and embedded in paraffin. Serial sections were cut at 4–5 μm thickness for staining. Hematoxylin–eosin (HE) and Masson's trichrome (MT) stains were used to evaluate epithelialization, inflammatory cell infiltration, granulation tissue thickness, and collagen organization. Immunohistochemical staining for CD34 was performed to evaluate angiogenesis, with microvessel density (MVD) quantified as the mean number of CD34 positive capillaries per high‐power field across five randomly selected non‐overlapping fields per sample. All histological and immunohistochemical analyses were performed by an experienced histopathologist who was completely blinded to the experimental design and treatment allocation. The only information provided was that the specimens were part of a wound healing study, ensuring unbiased interpretation of the results. No molecular assays such as qPCR or Western blotting were performed; mechanistic conclusions are based on histological and immunohistochemical observations.

### 2.10 Statistical Analysis

Data were expressed as mean ± standard deviation (SD) for continuous variables and median (interquartile range) for ordinal variables. Normality of continuous data was assessed using the Shapiro–Wilk test before choosing parametric or non parametric methods. Intergroup comparisons at each time point were analyzed using one‐way ANOVA with Tukey's post hoc test or the Kruskal Wallis test with Dunn's correction, as appropriate. Intragroup comparisons over time were assessed using repeated‐measures ANOVA or the Friedman test. Statistical significance was set at *p* < 0.05. Analyses were conducted using GraphPad Prism version 10.0.3 (GraphPad Software, San Diego, CA, USA) and IBM SPSS Statistics version 29.0 (IBM Corp., Armonk, NY, USA).

## 3 Results

### 3.1 Macroscopic Wound Closure

**FIGURE 1:**
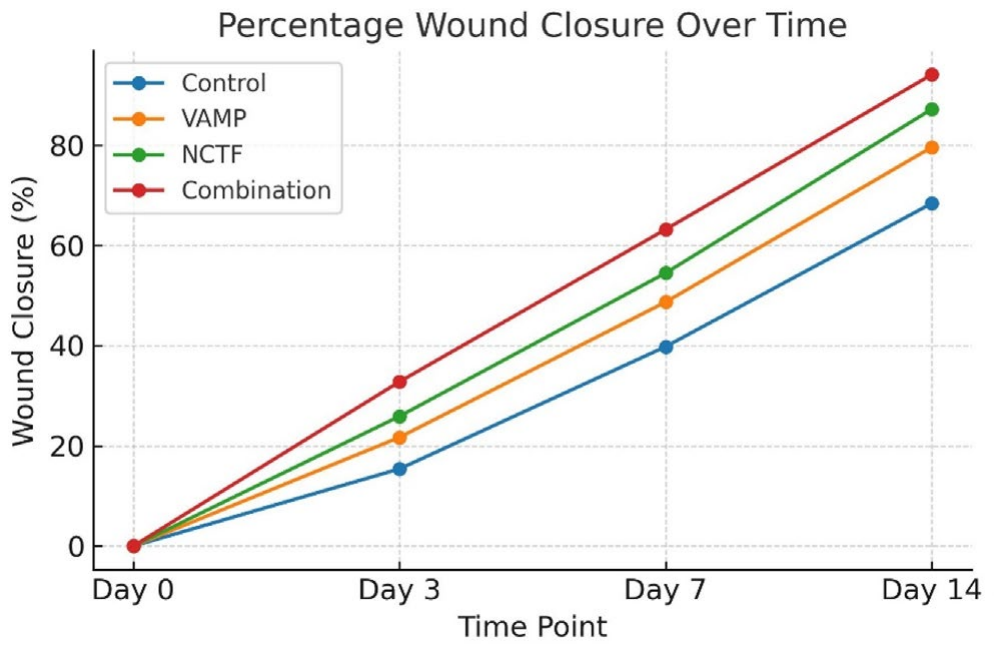
Percentage wound closure over time in control, VAMP, NCTF, and combination therapy groups. Wound area was measured at baseline (day 0), day 3, day 7, and day 14, and closure was expressed as a percentage relative to baseline. The combination therapy group demonstrated the fastest and most complete closure at all post injury time points, followed by NCTF, VAMP, and control.

All groups showed progressive reduction in wound area over the 14 day period, but the rate and extent of closure varied significantly (*p* < 0.001). By day 3, early contraction and epithelial migration were most evident in the combination group, followed by NCTF, VAMP, and control. At day 7, combination treated wounds had closed by 63.2% ± 4.8%, significantly greater than NCTF (54.5% ± 5.1%, *p* = 0.012), VAMP (48.7% ± 5.6%, *p* < 0.001), and control (39.8% ± 4.9%, *p* < 0.001). By day 14, the combination group achieved near complete closure (94.1% ± 2.3%), compared with 87.2% ± 3.0% for NCTF, 79.6% ± 3.8% for VAMP, and 68.4% ± 4.5% for controls (all pairwise *p* < 0.01) (Table 1 and Figure 1).

**TABLE 1:** Percentage wound closure at each time point (mean ± SD).

Day	Control (%)	VAMP (%)	NCTF (%)	Combination (%)
Day 0	0.0 ± 0.0	0.0 ± 0.0	0.0 ± 0.0	0.0 ± 0.0
Day 3	15.4 ± 2.1	21.7 ± 2.4	25.9 ± 2.6	32.8 ± 3.0
Day 7	39.8 ± 3.5	48.7 ± 3.2	54.5 ± 3.1	63.2 ± 3.6
Day 14	68.4 ± 4.1	79.6 ± 3.7	87.2 ± 3.4	94.1 ± 2.8

### 3.2 Histological Evaluation (Hematoxylin–Eosin)

Baseline sections confirmed full thickness skin disruption across all groups.

**FIGURE 2:**
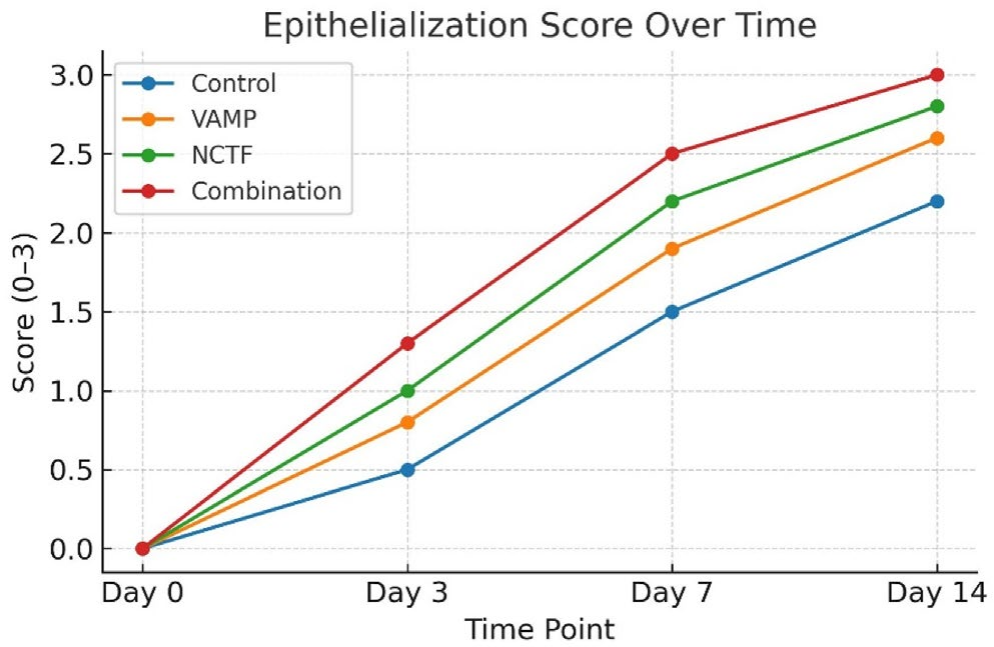
Epithelialization scores (0–3 scale) at each time point for all groups. Scores were assigned by blinded histological evaluation, with 0 indicating no epithelial coverage and 3 indicating complete, mature epithelialization. Combination therapy achieved the highest epithelialization scores at earlier time points.

By day 3, combination treated wounds exhibited reduced inflammatory infiltrate and early epithelial tongue formation compared with other groups. NCTF showed moderate re‐epithelialization with scattered polymorphonuclear cells, while VAMP and control wounds retained dense inflammatory infiltrates. At day 7, the combination group demonstrated nearly continuous epithelial coverage and well‐organized granulation tissue rich in fibroblasts and neovascular structures. NCTF achieved partial epithelial bridging, whereas VAMP wounds lagged, with control wounds showing persistent fibrin exudate. By day 14, the combination group displayed complete re‐epithelialization with minimal residual inflammation; NCTF wounds showed near complete coverage with mild chronic inflammatory cells, VAMP showed partial restoration, and control wounds retained patchy epidermal defects (Table 2 and Figure 2).

**TABLE 2:** Epithelialization scores (0–3 scale, median [IQR]).

Day	Control	VAMP	NCTF	Combination
Day 0	0 [0–0]	0 [0–0]	0 [0–0]	0 [0–0]
Day 3	0.5 [0–1]	0.8 [0–1]	1.0 [0–1]	1.3 [1–2]
Day 7	1.5 [1–2]	1.9 [1–2]	2.2 [2–3]	2.5 [2–3]
Day 14	2.2 [2–3]	2.6 [2–3]	2.8 [2–3]	3.0 [3–3]

### 3.3 Collagen Deposition and Organization (Masson's Trichrome)

**FIGURE 3:**
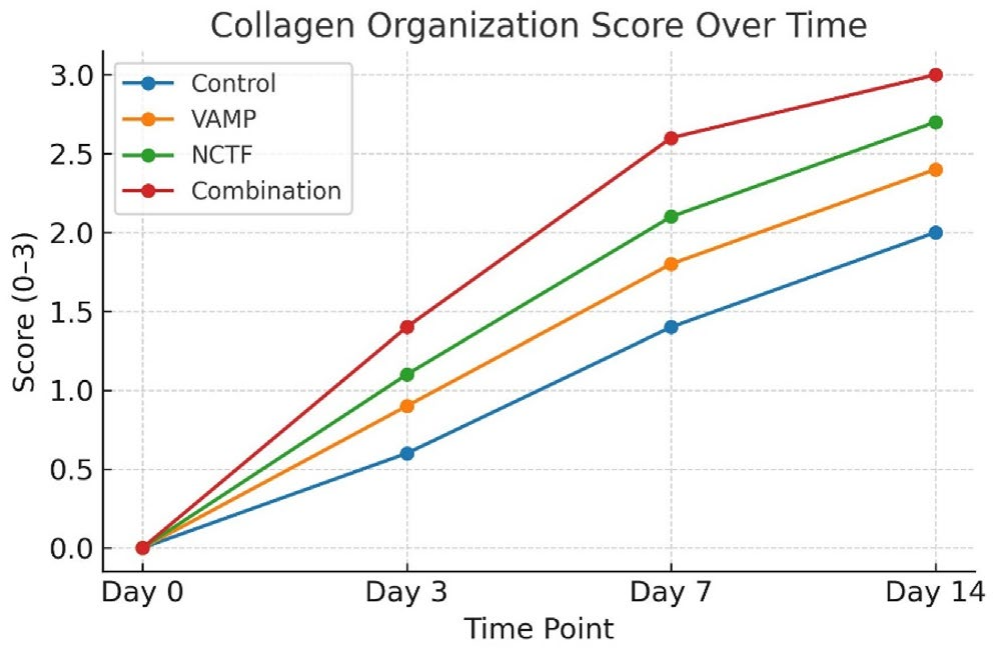
Collagen organization scores (0–3 scale) assessed by Masson's trichrome staining. Scores reflected the density, alignment, and maturity of collagen fibers. Combination therapy led to the most organized collagen deposition by day 14, followed by NCTF, VAMP, and control.

MT staining revealed a progressive increase in collagen deposition over time in all groups, with marked differences in fiber maturity and orientation. By day 7, combination therapy induced dense, well‐aligned collagen bundles occupying the wound bed, compared to less compact fibers in NCTF and loosely arranged fibers in. VAMP, and disorganized collagen in controls. By day 14, collagen in the combination group resembled normal dermis, with thick parallel bundles and minimal inter fiber spacing. NCTF treated wounds exhibited moderately organized bundles, VAMP wounds displayed thinner, irregular fibers, and controls showed loosely packed immature collagen (Table 3 and Figure 3).

**TABLE 3:** Collagen organization scores from Masson's trichrome (0–3 scale, median [IQR]).

Day	Control	VAMP	NCTF	Combination
Day 0	0 [0–0]	0 [0–0]	0 [0–0]	0 [0–0]
Day 3	0.6 [0–1]	0.9 [0–1]	1.1 [1–2]	1.4 [1–2]
Day 7	1.4 [1–2]	1.8 [1–2]	2.1 [2–3]	2.6 [2–3]
Day 14	2.0 [2–3]	2.4 [2–3]	2.7 [2–3]	3.0 [3–3]

### 3.4 Angiogenesis (CD34 Immunohistochemistry)

**FIGURE 4:**
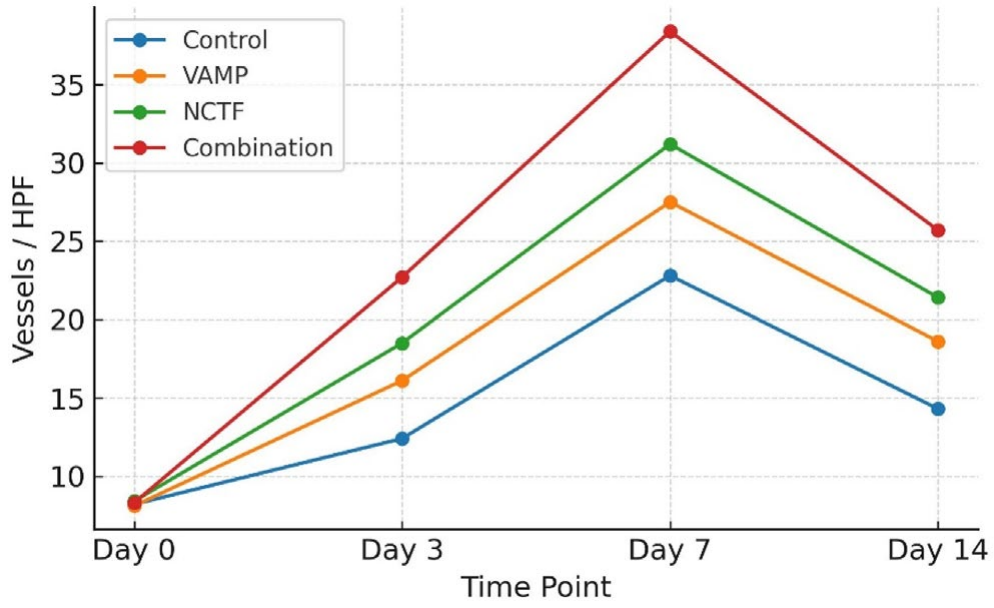
Microvessel density (MVD) quantified by CD34 immunohistochemistry at different time points after wounding. MVD was expressed as the mean number of CD34 positive capillaries per high power field (HPF) from five random fields per specimen. Combination therapy induced significantly greater angiogenesis, particularly at days 3 and 7, compared to all other groups.

CD34 staining demonstrated a peak in microvessel density (MVD) at day 7 in all groups, followed by a gradual decline by day 14. Quantitatively, MVD at day 7 was highest in the combination group (38.4 ± 3.1 vessels/HPF), followed by NCTF (31.2 ± 2.7), VAMP (27.5 ± 2.9), and control (22.8 ± 2.5) (all *p* < 0.01). At day 14, the combination group retained significantly higher MVD (25.7 ± 2.4) compared to NCTF (21.4 ± 2.1), VAMP (18.6 ± 1.9), and control (14.3 ± 1.8) (*p* < 0.05) (Table 4 and Figure 4).

**TABLE 4:** Microvessel density (CD34 positive capillaries/HPF, mean ± SD).

Day	Control	VAMP	NCTF	Combination
Day 0	8.2 ± 1.2	8.1 ± 1.3	8.4 ± 1.1	8.3 ± 1.2
Day 3	12.4 ± 1.8	16.1 ± 2.0	18.5 ± 1.9	22.7 ± 2.1
Day 7	22.8 ± 2.3	27.5 ± 2.2	31.2 ± 2.0	38.4 ± 2.4
Day 14	14.3 ± 1.9	18.6 ± 1.7	21.4 ± 1.8	25.7 ± 2.0

### 3.5 Histological and Immunohistochemical Observations

**FIGURE 5:**
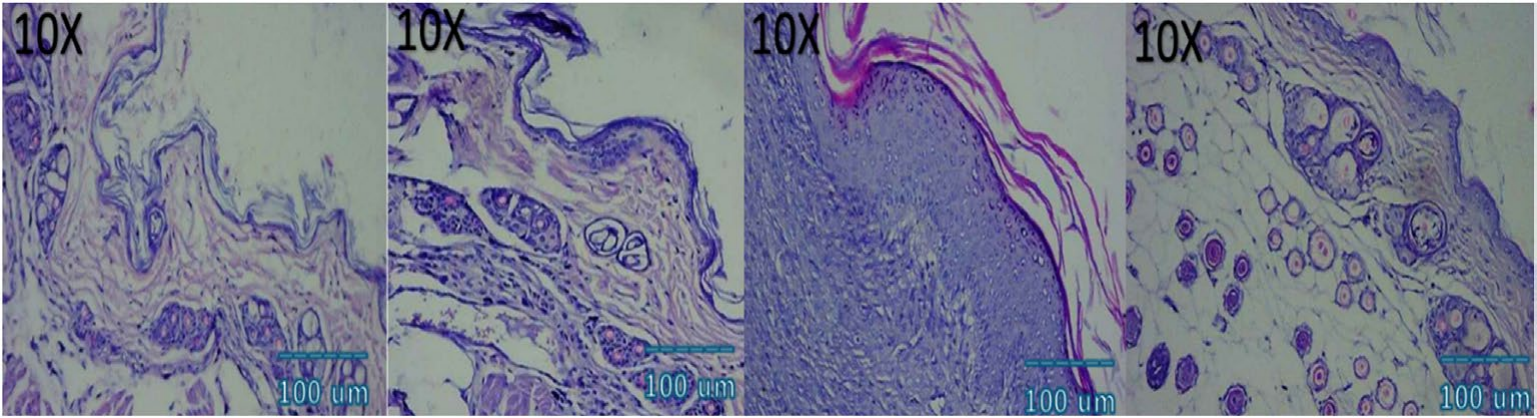
Photomicrograph of HE stain of NCTF group; from left to right: first picture (baseline), second picture (3 days), third picture (7 days), fourth picture (14 days).

**FIGURE 6:**
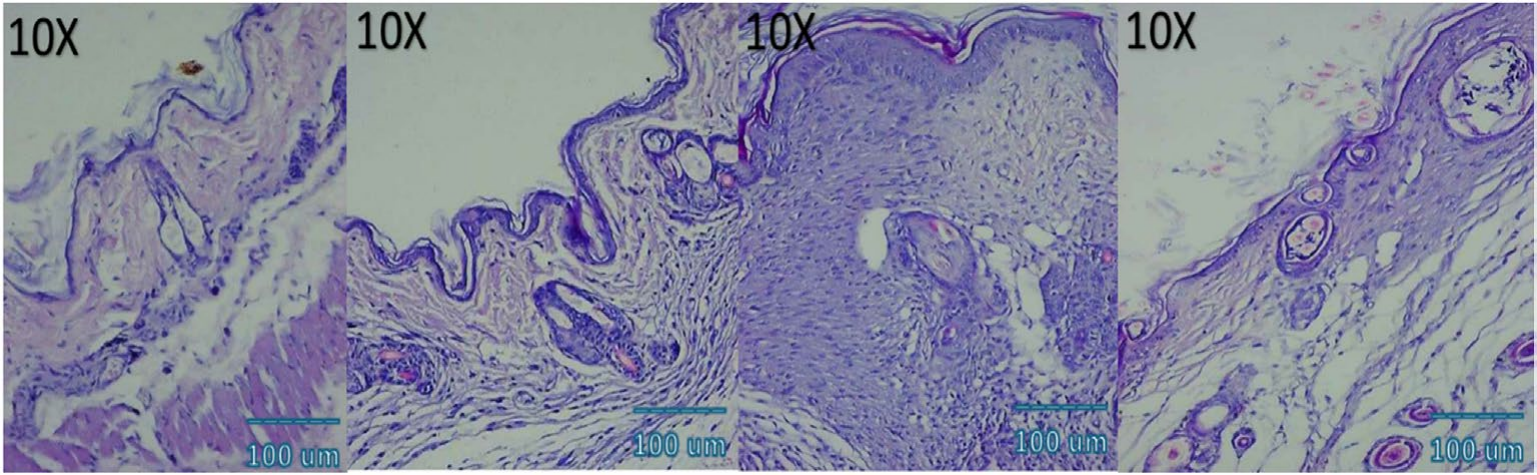
Photomicrograph of HE stain of VAMP group; from left to right: first picture (baseline), second picture (3 days), third picture (7 days), fourth picture (14 days).

**FIGURE 7:**
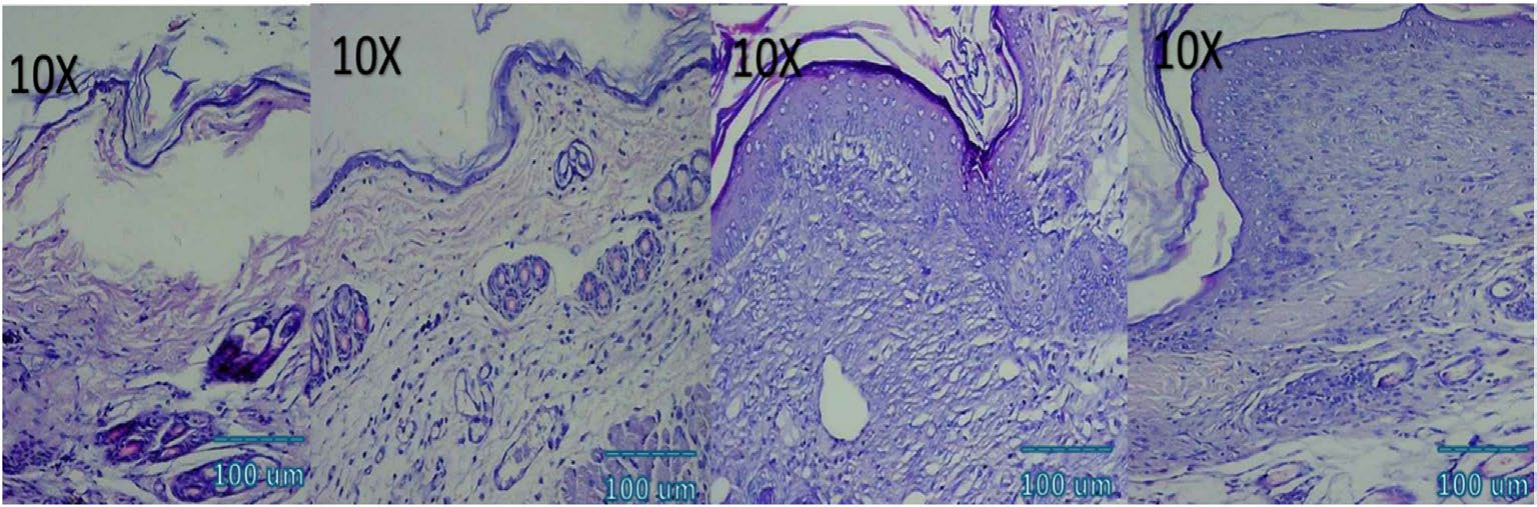
Photomicrograph of HE stain of NCTF + VAMP group; from left to right: first picture (baseline), second picture (3 days), third picture (7 days), fourth picture (14 days).

**FIGURE 8:**
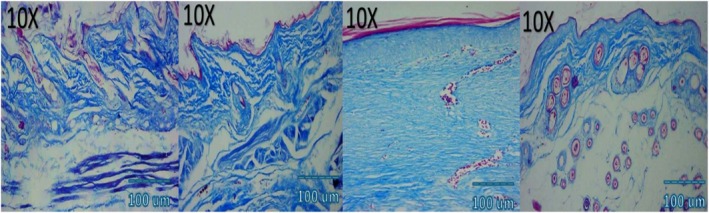
Photomicrograph of MT stain of NCTF group; from left to right: first picture (baseline), second picture (3 days), third picture (7 days), fourth picture (14 days).

**FIGURE 9:**
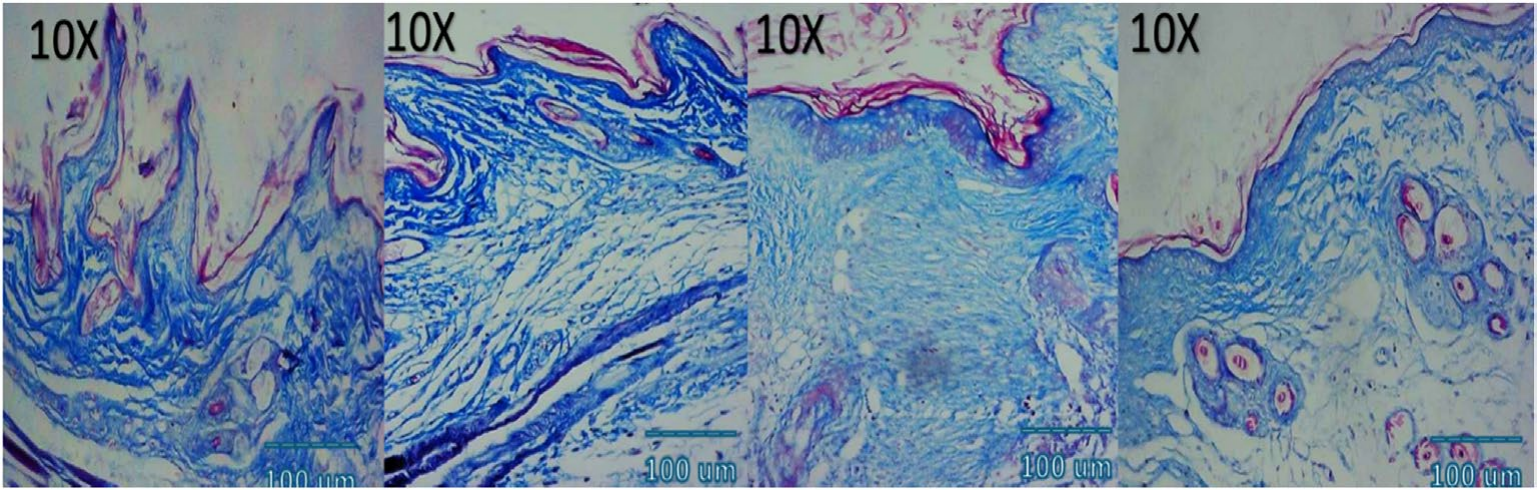
Photomicrograph of MT stain of VAMP group; from left to right: first picture (baseline), second picture (3 days), third picture (7 days), fourth picture (14 days).

**FIGURE 10:**
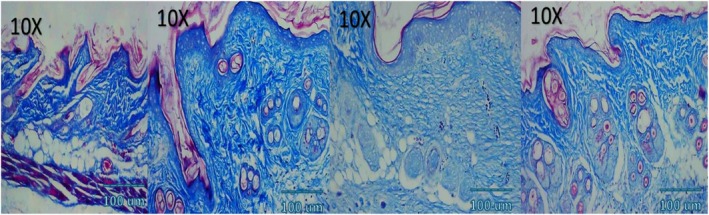
Photomicrograph of MT stain of NCTF + VAMP group; from left to right: first picture (baseline), second picture (3 days), third picture (7 days), fourth picture (14 days).

**FIGURE 11:**
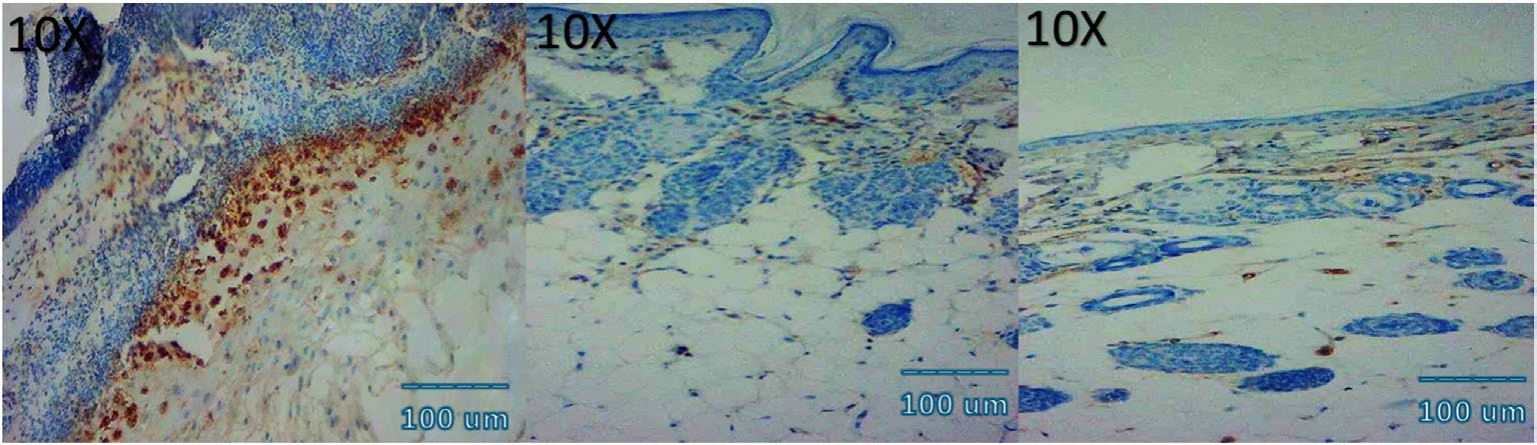
Photomicrograph of immunohistochemical CD34 stain of NCTF group; from left to right: first picture (3 days), second picture (7 days), third picture (14 days).

**FIGURE 12:**
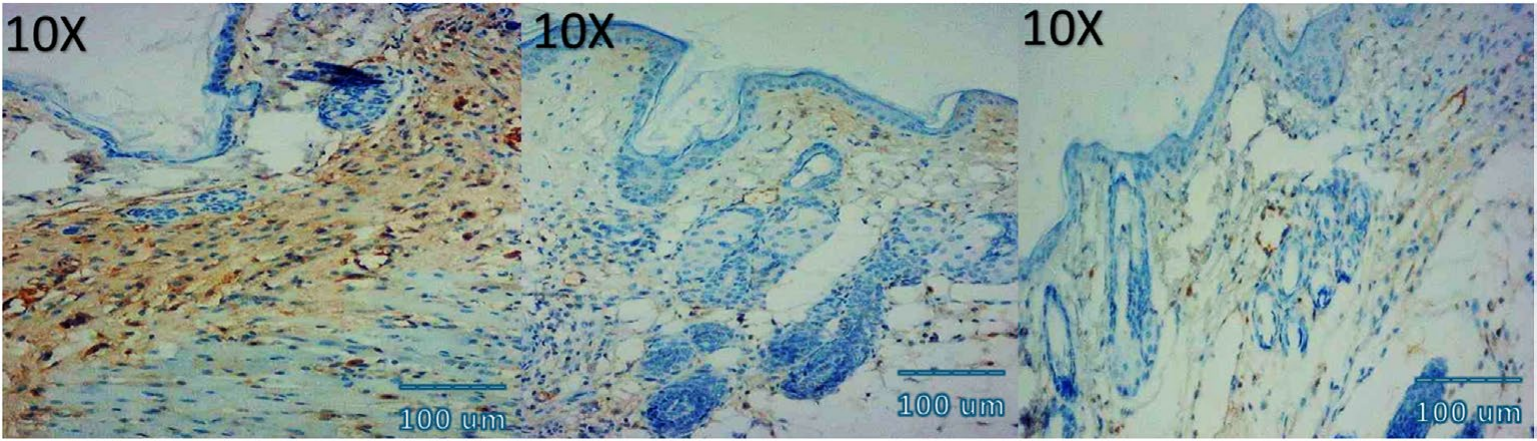
Photomicrograph of immunohistochemical CD34 stain of VAMP group; from left to right: first picture (3 days), second picture (7 days), third picture (14 days).

**FIGURE 13:**
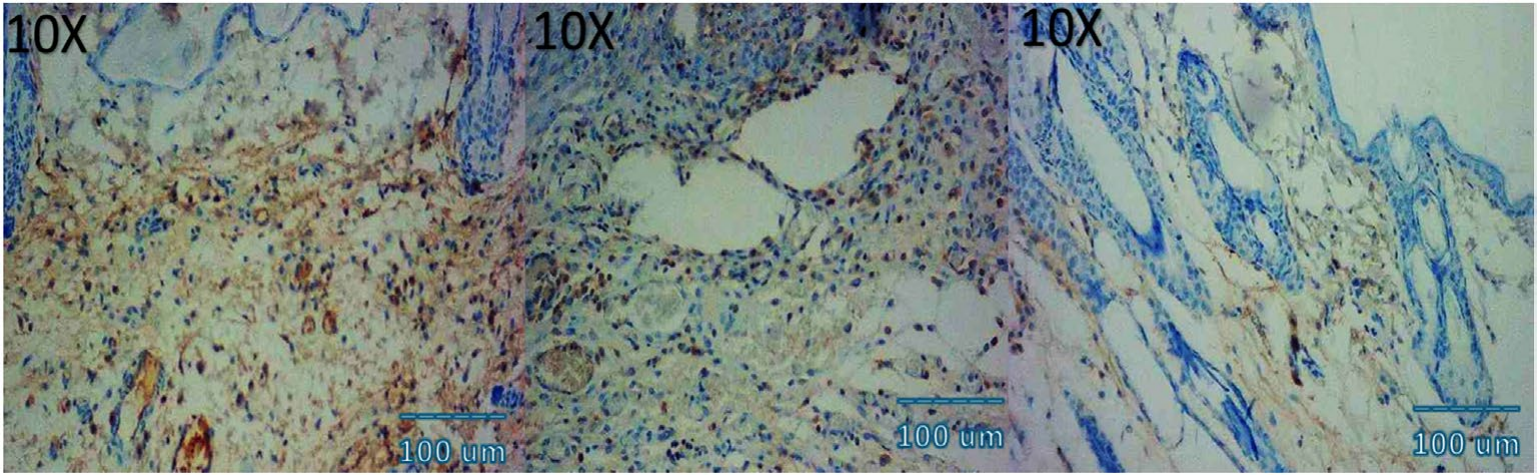
Photomicrograph of immunohistochemical CD34 stain of NCTF + VAMP group; from left to right: first picture (3 days), second picture (7 days), third picture (14 days).

Microscopic examination of HE stained sections revealed clear temporal and treatment‐dependent differences in wound healing across all groups. At day 3, control wounds exhibited wide gaps between wound edges, dense inflammatory infiltrates, fibrinous exudate, and minimal epithelial tongue formation. In contrast, NCTF and VAMP treated wounds showed reduced inflammation, early epithelial migration, and better‐organized granulation tissue, with the combination group displaying the most advanced epithelial bridging and cellular organization. By day 7, control wounds remained incompletely epithelialized, with loosely arranged granulation tissue and sparse capillary formation. NCTF treated samples demonstrated a continuous but immature epithelial layer, increased fibroblast proliferation, and moderate collagen deposition, while VAMP treated wounds exhibited abundant neovascular structures, as confirmed by CD34 staining. The combination group showed near complete epithelial closure, thicker granulation tissue, well oriented collagen bundles on MT staining, and the highest microvessel density among all groups. At day 14, control wounds still showed incomplete remodeling, irregular collagen alignment, and residual inflammatory cells. Both NCTF and VAMP groups exhibited more mature dermal architecture and reduced inflammation, though collagen fibers remained less organized than in the combination group. The latter displayed complete epithelial regeneration with a keratinized layer, densely packed and parallel collagen bundles, and a marked reduction in CD34 positive capillary density, consistent with vascular maturation and tissue remodeling (Figures 5, 6, 7, 8, 9, 10, 11, 12, 13).

## 4 Discussion

This study demonstrates that the combination of NCTF and PDRN containing VAMP accelerates healing of full thickness linear skin wounds in a standardized hamster model, outperforming either treatment alone. The observed improvements are likely due to complementary mechanisms: NCTF provides a nutrient rich milieu that enhances fibroblast proliferation, extracellular matrix synthesis (particularly type I collagen), dermal hydration, and keratinocyte migration [10, 14, 15], while PDRN acts through A2A adenosine receptor stimulation to promote angiogenesis via VEGF upregulation, support DNA synthesis in repairing cells, and modulate inflammation [11, 16, 17]. By targeting multiple pathways simultaneously, the combination therapy appears to create a more favorable microenvironment for tissue regeneration, producing additive or synergistic benefits [18, 19].

Analyzing the agents individually highlighted their distinct contributions: NCTF primarily improved collagen organization and epithelial coverage, whereas PDRN mainly enhanced microvascular formation. The combination therapy harnessed these complementary effects, promoting both structural and vascular aspects of healing. Early angiogenesis (days 3–7) and late phase collagen maturation (day 14) were particularly enhanced, supporting the concept of genuine biological synergy between these agents.

While these results are promising, caution is required in extrapolating to human clinical scenarios. Rodent skin heals predominantly through contraction, differs histologically from human skin, and this study used only healthy young male hamsters with a single injection. These findings should therefore be viewed as proof of concept, illustrating biological potential rather than immediate clinical applicability. Translation to clinical practice will require further validation in larger animal models, particularly those with impaired or chronic wounds, and carefully controlled human trials to determine optimal dosing, safety, and efficacy.

Several limitations should be acknowledged. The study's 14 day follow up allowed evaluation of inflammatory and proliferative phases but did not capture remodeling or scar maturation, important for assessing long term tensile strength and cosmetic outcomes. Only a single concentration and injection were tested, leaving the effects of repeated or delayed administration unexplored. Mechanistically, while histological analysis and CD34 immunostaining demonstrated increased angiogenesis and improved collagen organization, molecular pathways such as VEGF expression, collagen subtype regulation, and cytokine modulation were not directly measured; thus, mechanistic conclusions remain inferential. Finally, although blinded, macroscopic wound photography and planimetry may still introduce minor observer or technical bias.

Future research should extend follow up periods, incorporate impaired healing or chronic wound models, and integrate molecular analyses to confirm the pathways underlying the observed synergy. These studies will better define the translational potential of combination NCTF and PDRN therapy and provide a more comprehensive understanding of its mechanistic basis.

## 5 Conclusion

This experimental study demonstrated that the combination of NCTF and PDRN‐containing VAMP accelerated full thickness skin wound healing in a hamster model, surpassing the effects of either agent alone. The combined treatment enhanced wound closure, epithelialization, collagen organization, and angiogenesis, reflecting complementary and synergistic mechanisms. These findings are limited to early and intermediate phases of healing (up to 14 days) and support further investigation in larger animal models and controlled human studies before considering clinical application.

## Author Contributions

All authors have read and revised the manuscript and have contributed to all the work done in this study.

## Funding

The authors have nothing to report.

## Ethics Statement

This study was conducted in accordance with the ARRIVE Guidelines for animal use. Ethical approval was obtained from an Institutional Animal Care and Use Committee.

## Consent

The authors have nothing to report.

## Conflicts of Interest

The authors declare no conflicts of interest.

## Data Availability

The datasets generated and/or analyzed during the current study are available from the corresponding author on reasonable request.
